# Effect of Sugarcane Bagasse Content and Modification on the Properties of Sugarcane Bagasse/Poly(lactic Acid) Biocomposites

**DOI:** 10.3390/molecules30071583

**Published:** 2025-04-02

**Authors:** Youxue Peng, Wen Lei, Wangwang Yu, Yong Chen

**Affiliations:** 1 College of Science, Nanjing Forestry University, Nanjing 210037, China; 2 School of Mechanical Engineering, Nanjing Vocational University of Industry Technology, Nanjing 210023, China

**Keywords:** poly(lactic acid), sugarcane bagasse, biocomposite, inorganic salt

## Abstract

In this study, poly(lactic acid) biocomposites were prepared from sugarcane bagasse (SB) via extrusion and injection molding. The effects of the content and inorganic salt modification of SB on the properties of the biocomposites were investigated. The results showed that the incorporation of SB reduced the biocomposites’ mechanical strength and modulus as well as thermal stability but increased their crystallinity, hydrophobicity, and water absorption compared with neat PLA. Among all the biocomposites, the sample containing 30 wt % SB(SB-30/PLA) had the best comprehensive performances, with tensile strength, tensile modulus, flexural strength, and crystallinity values of 31.78 MPa, 219.49 MPa, 53.25 MPa, and 16.8%, respectively. After SB modification with Na_2_SO_4_ and MgSO_4_, the increased interfacial adhesion led to a considerable improvement in reinforcement and increases in the flexural strength, flexural modulus, impact strength, and crystallinity of SB-30/PLA; furthermore, the biocomposite became more thermally stable and hydrophobic and contained much less water. In conclusion, SB-30/PLA, especially after MgSO_4_ modification, is an ideal degradable biocomposite for applications in packaging, decoration, and other areas.

## 1. Introduction

The increasing requirement for more environmentally sustainable products has motivated the replacement of “fossil carbon” with “renewable carbon”. As typical representatives of “renewable carbon”, biodegradable polymers can be completely degraded into CO_2_ and H_2_O by microorganisms after disposal. This process makes it possible for the products to undergo ecological recycling, effectively avoiding pollution problems associated with the use of petroleum-based plastics [1,2].

Among these biodegradable polymers, polylactic acid (PLA) is produced and used in the largest quantities [3] and thus plays a leading role in the sustainable plastic economy [4]. Being derived naturally from agricultural products, such as corn and wheat [5,6], PLA is biodegradable and has numerous excellent physico-mechanical characteristics, including outstanding melt processing ability, high strength, a low thermal expansion coefficient, low elongation at break, and no cracking into large pieces [4,6,7]. However, its inherent drawbacks, especially its much higher cost compared with general-purpose plastics such as polyethylene and propylene, have hampered its wider practical application [8,9].

Natural fibers are abundantly available in nature and have a low cost, low specific weight, and good specific mechanical properties. Their rapid renewability has made them potential alternative composite materials [5,7]. To achieve different objectives, a number of polymers have already been complexed with various natural fibers, such as wood fiber [10,11], bamboo [12,13], jute [1,14], hemp [15,16], sisal [17], banana fiber [18,19], coconut fiber [20], coffee husk [21], rice husk [22], wheat husk [22], and straw fibers (sorghum, rice, corn, and soybean) [23] as well as their hybrids [24]. Sometimes, these polymers are also reinforced in the form of cellulose nanocrystals [25]. Most of these natural fibers have also been adopted as reinforcements in PLA-based composites.

Sugarcane bagasse (SB), a residue that is widely manufactured in high proportions in the sugar-making industry and is one of the most important natural fibers, is cheap, abundant (with an average annual production of 675 million tons of bagasse as a waste material [26]), and biodegradable. However, its utilization rate is not very high, as a large amount of SB is often burnt, which not only pollutes the environment but also creates resource waste. Therefore, the reuse of sugarcane bagasse as a resource is essential. One of the most common methods of making SB reusable is preparing composites made of an SB and polymer matrix. Sugarcane bagasse is composed of cellulose (40–50%), hemicelluloses (25–35%), and lignin (25–28%) [27]. The high concentration of cellulose, which has a crystalline structure, allows SB to perform optimally as a composite reinforcement [26], and some publications have reported on this topic. Patil et al. [27] studied a compression-molded composite made from SB and hybrid resin (a combination of epoxy and natural resin, such as dammar, pine, and cashew nutshell liquid). The mechanical properties, soil burial weight loss, microbial degradation, and CO_2_ evolution of the composite were found to differ with the type of natural resin and the mixing ratio of SB–epoxy resin–natural resin. Anggono et al. [28] prepared an SB/PP composite using wood fiber (WF) as a reference filler. They concluded that SB could reinforce PP similarly to when other natural fibers were used, specifically that SB- and WF-reinforced composites had similar properties; both composites had enhanced impact resistance compared with that of the neat matrix. As a hydrophilic natural fiber, SB was incompatible with the hydrophobic polymer matrix; poor interfacial adhesion caused the composite material to have poor physico-mechanical properties. To overcome this defect, Vidyashri et al. [29] chemically modified SB via alkaline, permanganate, and phosphoric acid treatments and evaluated the mechanical properties of the untreated and treated fiber-reinforced epoxy composites. After treatment, both the Young’s modulus and tensile strength of the sample improved, and the permanganate treatment had the greatest positive effects on the mechanical properties. The Young’s modulus and tensile strength of the sample increased by 22.8% and 40.6%, respectively, compared with those of pure epoxy resin. Lalta et al. also observed that alkaline treatment could improve the mechanical properties of an SB/epoxy composite [30]. In addition, they found that both the variety and the length of the fiber had significant effects on the properties.

In order to compensate for the disadvantages of PLA—particularly to reduce its high costs, resource waste, and environment pollution—as well as to make full use of SB, in this study, a fully biodegradable biobased composite made from PLA and sugarcane bagasse fibers was prepared, and its physico-mechanical property profile was explored systematically for the first time; additionally, a novel method of modifying SB using inorganic salt was introduced, with the aim of examining the impact of SB content on the biocomposite’s properties and significantly improving its comprehensive performance after modification. The practical relevance of the results is also discussed briefly at the end of this paper.

## 2. Results and Discussion

### 2.1. Effects of SB Content on the Properties of the Biocomposites

#### 2.1.1. Effects of SB Content on the Mechanical Properties of the Biocomposites

**Figure 1:**
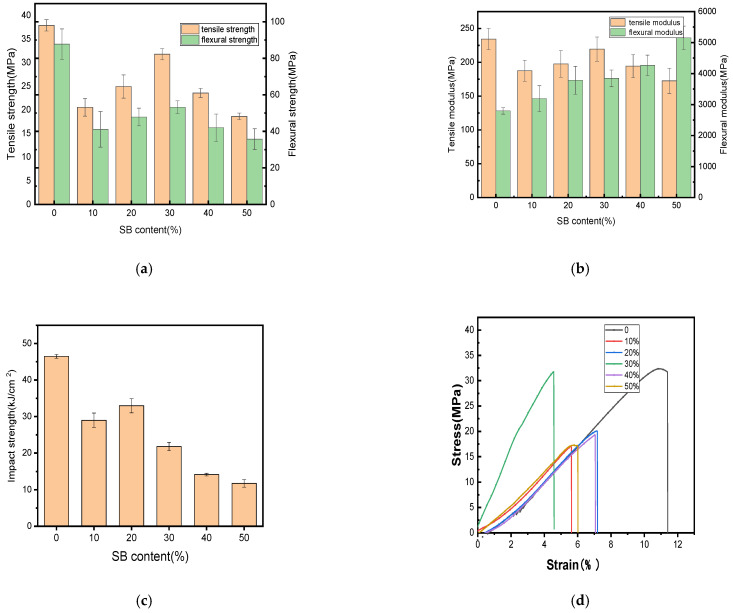
Mechanical properties of the PLA and SB/PLA biocomposites: (**a**) tensile and flexural strength; (**b**) tensile and flexural modulus; (**c**) impact strength; (**d**) stress–strain curve.

**Figure 2:**
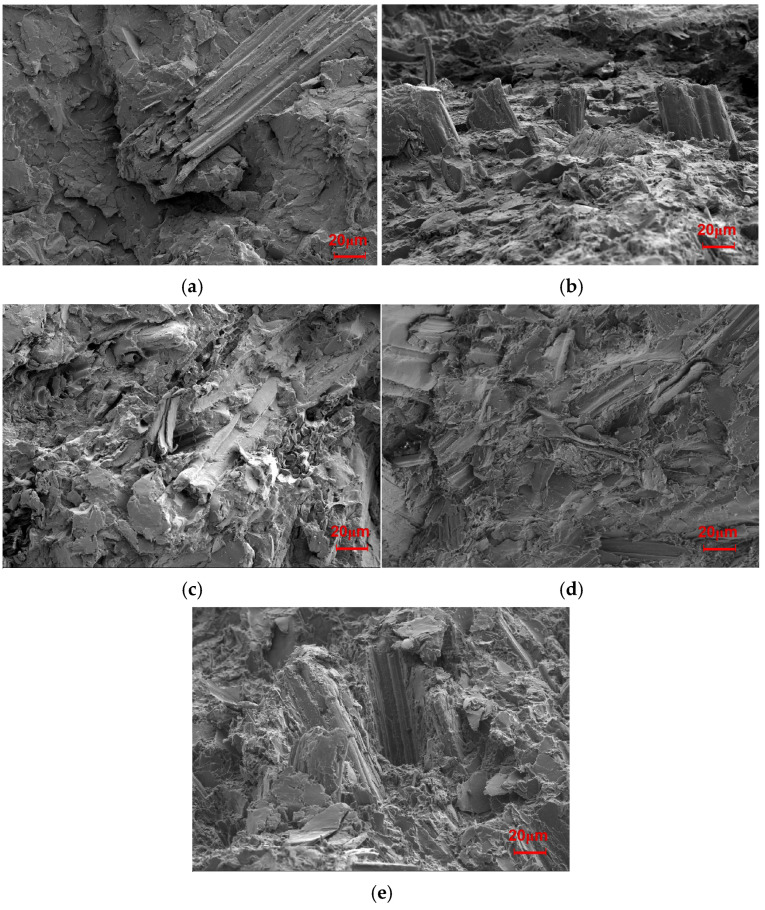
SEM images of the fractured section of the SB/PLA biocomposites: (**a**) SB-10/PLA, (**b**) SB-20/PLA, (**c**) SB-30/PLA, (**d**) SB-40/PLA, (**e**) SB-50/PLA.

The changes in the mechanical properties after the tests performed in this study are shown in Figure 1. Additionally, the fracture surfaces of the biocomposites with different SB loadings were further explored to determine their breaking mechanism, as illustrated in Figure 2.

Figure 1a,b demonstrate that the incorporation of SB reduced the tensile and flexural strengths of PLA. Similar results have been reported on some other natural fiber/polymer composite materials, such as WF/PP [31], WF/ABS [32], and coffee ground/PBAT [33]; this reduction might be due to some defects inside the composite and the poor interfacial compatibility between the matrix and the filler. The results of the impact strengths in Figure 1c revealed that the SB/PLA biocomposites exhibited lower impact strengths than their parent polymer, once again due to its immiscible character.

Figure 1 illustrates the tensile strength, tensile modulus, flexural strength, flexural modulus, and impact strength of the specimen composed solely of PLA, with average measurements of 37.89 MPa, 234.36 MPa, 87.80 MPa, 2795.32 MPa and 46.47 kJ/m^2^, respectively. As the content of SB increased, all the mechanical properties except for the flexural modulus increased first and then decreased; this tendency was in accordance with the tensile test results of nanocellulose/poly(vinyl alcohol) (PVA) composites [34]. In this study, SB-30/PLA had the greatest tensile strength, flexural strength, and tensile modulus, which were 31.78 MPa, 53.25 MPa, and 219.49 MPa, respectively, which may be due to the combined actions of the two opposite roles played by SB in the composites. On one hand, the poor interfacial compatibility between SB and PLA because of their polar differences produces many defects in the composite, leading to poorer mechanical properties [20,29]; on the other hand, SB, as a natural fiber, could reinforce the composite [35]. When PLA was compounded with SB via a melt blending process, hydrogen bonds formed between the polar hydroxyl groups (-OH) from cellulose and hemicellulose in SB and the carboxyl groups (-COO-) in PLA, which became the main force between the reinforcement and the matrix. The SB particles were embedded in the PLA matrix, forming a physically entangled structure that enhanced interfacial bonding.

When a small amount of SB was used, the number of fibers was not sufficient for them to be fully distributed in the matrix, and thus, their ability to provide reinforcement was limited. As evidenced from Figure 2a, a very small amount of SB was observed when the SB loading was 10%. In Figure 2b, more SB fibers appeared, but not enough yet to be homogeneously distributed in the matrix. As a result, the mechanical properties worsened to a greater extent; when the dosage of SB was increased to 30 wt %, a lot of SB was found to be wrapped in the PLA (Figure 2c), and enough fiber accumulated to form a continuous reinforcement net, thus increasing the mechanical properties compared with those of SB-10/PLA and SB-20/PLA. When more SB was used (Figure 2d,e), however, the uneven dispersion of high-content SB led to the formation of aggregates and increased the porosity of the composites. In addition, PLA failed to coat the SB particles, resulting in poor bonding between the SB particles and the polymer and obvious debonding of SB from the matrix, as shown in Figure 2d and especially in Figure 2e; consequently, the stress could not be transferred from SB to PLA effectively, and the mechanical properties worsened [32].

The ductility of the SB/PLA biocomposite reduced significantly with the incorporation of SB, as demonstrated in Figure 1d. Similar results had also been reported in a previous study [32]. In detail, the percentage of the elongation at break of PLA was only 11.37%, and all the composites had lower percentages of elongation at break than PLA, meaning that all these samples would break in a brittle manner, which was consistent with the SEM observation from Figure 2.

#### 2.1.2. Effects of SB Content on the Thermal Stability of the Biocomposites

**Figure 3:**
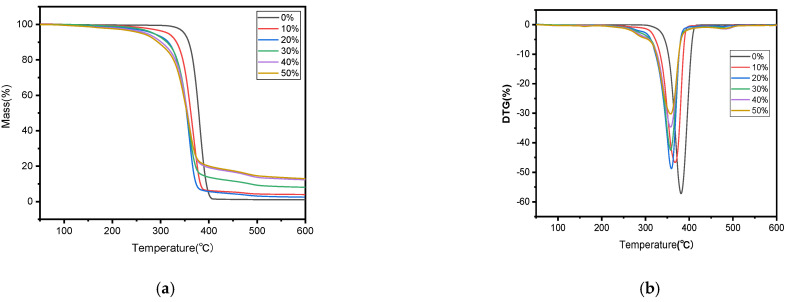
TGA (**a**) and DTG (**b**) curves of PLA and SB/PLA biocomposites.

The thermal stability variations among the biocomposites containing different amounts of SB were examined through TGA and DTG curves, as revealed in Figure 3. The similarity in the main components of the samples resulted in thermal gravimetric curves that were basically the same.

**Figure 4:**
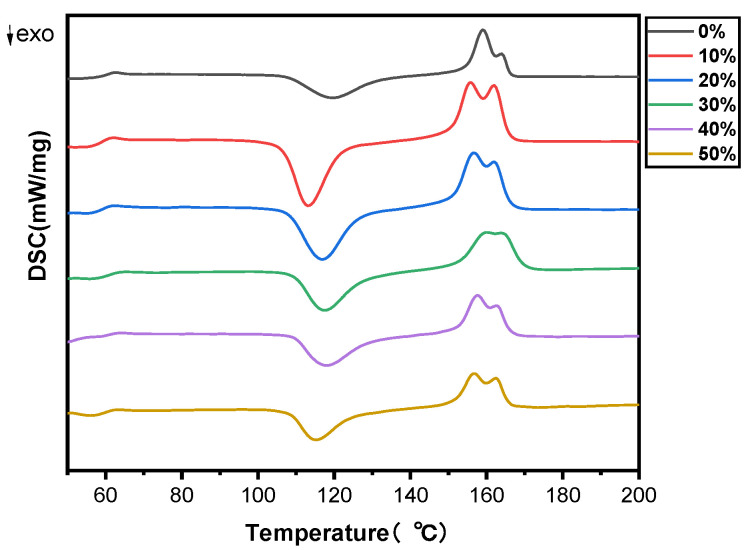
DSC thermograms of PLA and SB/PLA biocomposites.

In Figure 3a, it is shown that the mass of each sample dropped by a tiny bit within the temperature range of 30–150 °C, and this drop became more distinct when more SB was introduced, likely due to the evaporation of water molecules adsorbed by the polar groups in the sample. The main thermal degradation happened between 200 and 400 °C, during which more than three quarters of the total mass underwent thermal degradation; the relevant T_i_ and T_p_ values are outlined in Table 1. This stage was characterized by the breakage of the molecular chains of hemicellulose and cellulose, and the partial breakage of those of lignin [12] and PLA under high temperatures. From Figure 4, a third degradation can be found at around 500 °C, but this degradation rate was very low, perhaps caused by the degradation of residual lignin and PLA in the sample. In comparison with PLA, all the SB/PLA biocomposites exhibited a significant decrease in both T_i_ and T_p_ (Table 1), indicating that the addition of SB into PLA primarily led to composites characterized by poorer thermal properties than that of neat PLA processed under similar conditions. This might be ascribed to the poor thermal stability from additives of natural fibers, which changed the decomposition temperature of the composites. Similar conclusions had also been drawn on some other natural fiber/polymer composites in earlier studies [35]. In terms of the effects of SB content on the thermal stability of the samples, as seen in Table 1, both the T_i_ and T_p_ values decreased monotonically with SB content, indicating a gradual decrease in the thermal stability of the samples with an increasing dosage of SB. Further investigation revealed that marked drops in T_i_ and T_p_ happened when the load of SB in the samples increased from 0 to 20 wt % and when the SB content rose from 20 wt % to 30 wt %; however, its T_i_ and T_p_ values did not change much, meaning that SB-30/PLA had almost the same thermal stability as that of SB-20/PLA.

#### 2.1.3. Effects of SB Content on the Melt and Crystallization Behavior of the Biocomposites

The melting and crystallization properties of PLA and SB/PLA biocomposites were analyzed quantitatively via the DSC technique. The DSC thermograms of pure PLA and SB/PLA biocomposites in the second heating cycle are presented in Figure 4 and Table 2. As seen in Figure 4, the DSC curves of all the samples were quite similar to one another, indicating the negligible effect of SB on the melt and crystallization behavior of PLA. From Table 2, the glass transition temperature (T_g_) of PLA was 58.9 °C, which agreed with the value of 58.4 °C reported by Yue et al. [35] and that around 60 °C reported by Aliotta et al. [16], and almost no change was observed in the T_g_ value of the PLA matrix with the addition of SB up to 50 wt %. The cold crystallization temperature (T_cc_) and melting temperature (T_m_) values of PLA were found to have dropped when SB was added, which implies that SB acts as a nucleating agent for the cold crystallization of PLA [25], the uniformity of the PLA chain segment was broken [36], and the chain motion of PLA was facilitated by SB. The decrease in the Tcc of a polymer with the addition of natural fiber was also shown in the study conducted by Ramirez et al. [5]. Nevertheless, both the T_cc_ and T_m_ values of the SB/PLA biocomposites increased first and then decreased, and SB-30/PLA had the greatest T_cc_ value of 128.20 °C and a T_m_ value of 159.01 °C. These results are consistent with those from upcoming crystallinity tests.

Additionally, as seen in Table 2, PLA had enthalpy changes during cold crystallization of 38.22 J/g, which was marginally lower than that for the melting enthalpy (39.53 J/g); consequently, its crystallinity, calculated from the enthalpies, was as low as 1.39%, suggesting that the PLA in the composites was partially crystallized. With the increase in SB content in the composites, the enthalpy change upon the melting of PLA increased at first and then decreased, while that in the cold crystallization during the glass transition decreased considerably. On one hand, this may have been due to the dilution of the PLA concentration with the incorporation of a higher loading of SB; on the other hand, it meant that a substantial portion of the amorphous region was replaced with a crystalline region during the cooling process [35]. When crystallinity was taken into consideration, each biocomposite exhibited a much greater value than PLA, perhaps due to the nucleation effect of SB on PLA [37]. In addition, the X_c_ value increased first and then decreased. Similar to the changing trend of T_cc_ and T_m_ values, SB-30/PLA had the greatest X_c_ value of 16.8% among all the biocomposites, while the X_c_ values of SB-40/PLA and SB-50/PLA dropped to 11.4% and 7.99%, respectively, suggesting that too much SB inhibited further improvements in crystallization; in this situation, SB could act as a nuclear agent, and at the same time, too much fiber would limit the molecular chain movement of PLA and consequently hinder the crystallization behavior of PLA. The positive and negative effects resulted in increased crystallinity from PLA, but not as much as that in SB-30/PLA.

#### 2.1.4. Effects of SB Content on the Hydrophilicity of the Biocomposites

**Figure 5:**
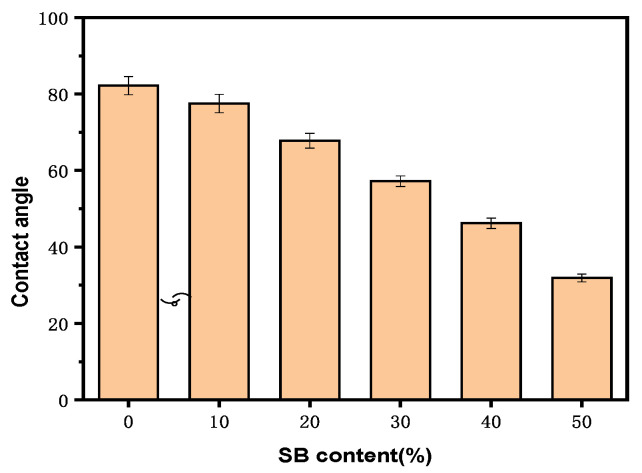
Water contact angles of the PLA and SB/PLA biocomposites.

There were concerns that fiber materials lead to excess water uptake as a result of their hydrophilic nature and poor interfacial interactions with hydrophobic polymers such as PLA. The effect of SB content on the water contact angle for the samples is shown in Figure 5.

All the samples had contact angles smaller than 90°, indicating that their surfaces were all hydrophilic. Specifically, the water contact angle on the surface of the PLA sample was 83.2°; after the introduction of SB, the surface wettability of the samples was enhanced. A gradual decrease in contact angle was observed in the composite materials with increasing SB content, and the contact angle on the surface of SB-10/PLA was 75.5°, while that on the surface of SB-50/PLA dropped to 32.3°. This decrease was probably due to the hydrophilicity of the SB powder.

#### 2.1.5. Effects of SB Content on the Water Absorption of the Biocomposites

**Figure 6:**
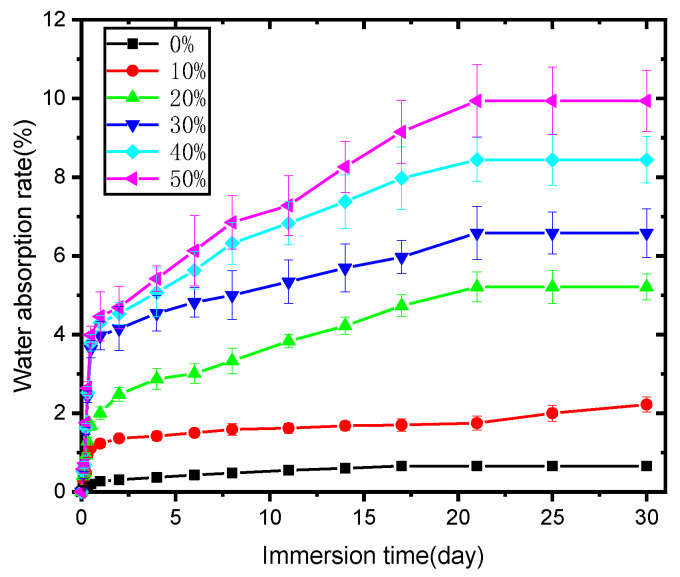
Water absorption (%) of the PLA and SB/PLA biocomposites with immersion time.

Figure 6 reveals the water absorption behavior of PLA and its biocomposites; PLA itself showed minimal water absorption, and immersion time had little effect on it. The water percentage absorbed by PLA was always less than 1%, although it increased with immersion time. As for the biocomposites, they absorbed much more water than PLA at any immersion stage and showed gradually increased water absorption percentages with soaking time; furthermore, a greater content of SB in the biocomposite led to more water absorption by the sample, perhaps due to the following reasons. SB, as already mentioned, is mainly composed of cellulose, lignin, and hemi-cellulose [27] and thus is inherently hygroscopic. A higher dosage of SB would thus result in an expected increase in water absorption. Additionally, lignocellulosic filler content in biocomposites might result in differing particle distributions, amounts of micro-voids, and interfacial compatibility with the polymer, potentially causing differences in the rate of water absorption in the composite samples.

### 2.2. Effects of SB Modification on the Properties of the Biocomposites

From the above discussions, SB-30/PLA was found to have the best comprehensive properties among all the biocomposites. It had the greatest tensile strength, flexural strength, tensile modulus, and crystallinity, and it had almost the same thermal stability as SB-20/PLA but was much more thermally stable than the other biocomposites.

When compared with pure PLA, however, the biocomposite also had some weaker properties. To reduce the negative impact of the addition of SB on the properties of PLA, some modifications to the fiber were necessary. Many efforts to treat natural fibers to improve the interaction between the reinforcing material and the matrix in composite materials have been made by researchers, with the most typical traditional methods being alkali [14,19] and coupling agent [5,7] treatments. These modifications can generally improve the comprehensive performance of natural fiber/polymer composites, but they also have some limitations. For alkali treatments, compounds with strong alkalinity, such as NaOH or KOH, are adopted, so a special container needs to be used to resist corrosion from hydroxide; in addition, post-treatment of the waste liquid has met with a little difficulty. For coupling agent treatments, the agent itself is expensive; furthermore, the used amount is not always sufficient to cover the fibers homogeneously. To solve this problem, inorganic salts were introduced in this research for the first time to modify sugarcane bagasse fibers, with the relatively greater ion strength from the salt promoting dissipation of the fibers; furthermore, the interaction between the natural fiber and matrix was enhanced due to the formation of the complex compound between the metal cation in the salt and the oxygen atom in the hydroxyl groups in the biomass material.

Next, SB-30/PLA was used to investigate the effect of inorganic salt modification on the properties of the biocomposites.

#### 2.2.1. FTIR Analysis and SEM Observations of the Filler SB

**Figure 7:**
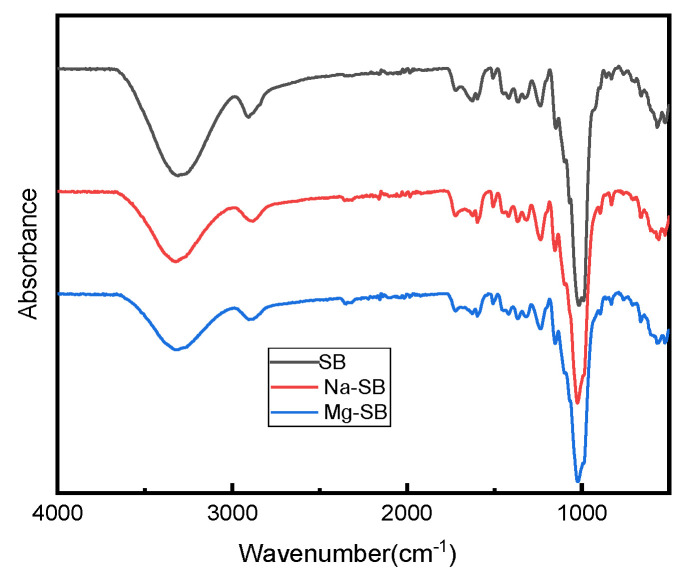
FTIR spectra of SB, Na-SB, and Mg-SB.

Figure 7 shows the FT-IR spectra of the original and modified SB.

The spectra of both the original and modified SB showed several common peaks that were attributed to SB. The strong absorption peaks around 3294 cm^−1^ correspond to the stretching of the hydroxyl groups (-OH), which belong to cellulose and lignin [38]; the absorption peaks that occurred at around 2890 cm^−1^ correspond to the vibration from C–H symmetrical stretching in the polysaccharides [39]; the typical absorption peaks at around 1611 cm^−1^ and 1335 cm^−1^ are attributed to the aromatic skeleton vibrations in lignin [39]; the peak at 1232 cm^−1^ corresponds to the coupling frequency of C-O and O-H; the peak around 1159 cm^−1^ is attributed to C–O–C asymmetrical stretching in cellulose [39]; the peak around 1080 cm^−1^ can be assigned to the in-plane deformation vibration of the C–H linkage of lignin [38]; and the characteristic signals around 670 cm^−1^ represent C–OH out-of-plane bending in cellulose [39].

Compared with the original SB fiber, no new absorption peaks appeared for modified SB fibers, showing that inorganic salt modification did not destroy the basic chemical structure of SB. However, a visible difference between the spectra of pure and modified SB fibers could be found at the peaks around 3294 cm^−1^. The peaks were weaker in the spectra for the modified SB than in that for the original SB fiber; this change might prove effective in reducing the number of hydroxyl groups contained in lignocellulosic waste [40] after SB modification. Accordingly, the hydrophilicity of SB decreased, a result consistent with the results from subsequent water contact angle testing.

**Figure 8:**
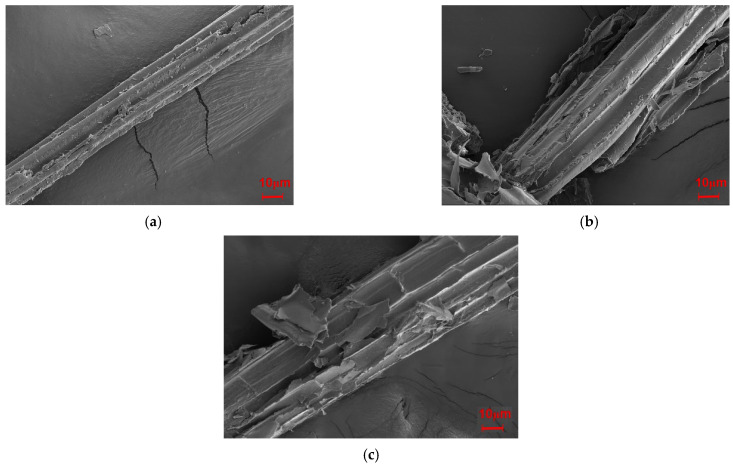
SEM images of the SB fibers: (**a**) unmodified SB, (**b**), Mg-SB, and (**c**) Na-SB.

The effects of modifying the microstructures of SB were observed via SEM; as illustrated in Figure 8, the modifications made the surface of the fiber rougher and the diameter larger, especially after the MgSO_4_ treatment. This significant increase in the diameter of the fiber meant that the inorganic salt modification loosened the fibers.

From the FTIR analysis and SEM observations, the physical structure of SB was found to have been modified, while its chemical components remained almost unchanged following treatments with inorganic salt.

#### 2.2.2. Visual Appearances of SB and Biocomposites

**Figure 9:**
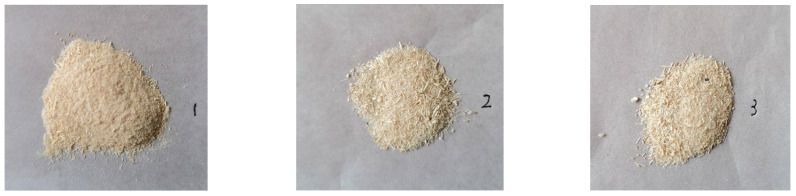
Visual appearance of the SB fibers. From **left** to **right**: SB, Mg-SB, and Na-SB.

**Figure 10:**
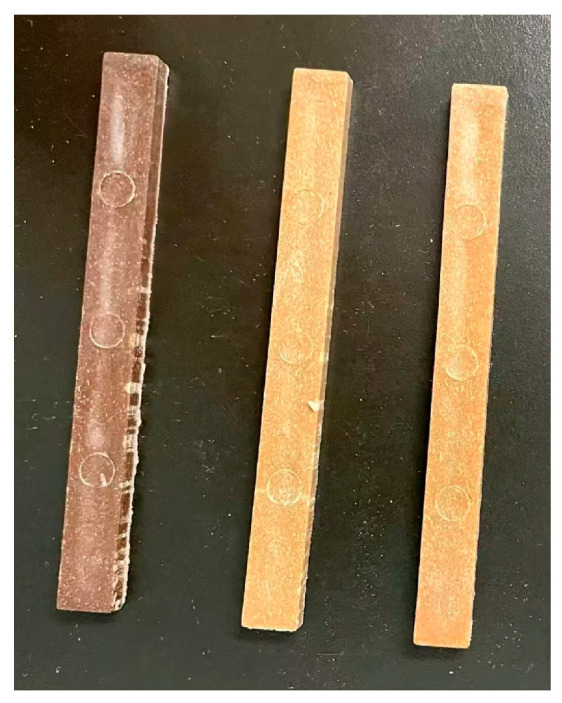
Visual appearance of the biocomposites. From **left** to **right**: SB-30/PLA, Mg-SB/PLA, and Na-SB/PLA.

The visual appearances of the SB and biocomposites are shown in Figure 9 and Figure 10, respectively. From Figure 9, the surface color of the fiber was found to have become lighter after the modification, leading to a lighter color in the modified samples, as shown in Figure 10. Specifically, the unmodified sample of SB-30/PLA had a brown appearance, while the surfaces of Mg-SB/PLA and Na-SB/PLA became light yellow. This alteration in the surface color indicates that the three samples’ compositions produced different optical effects, which could prove a change in the physical structure of SB to some extent; in other words, both the inorganic salts MgSO_4_ and Na_2_SO_4_ can modify SB effectively.

#### 2.2.3. Effects of SB Modification on the Mechanical Properties of the Biocomposites

The influences of SB modification on the flexural and impact properties of the biocomposites are presented in Table 3.

As seen in Figure 1, the flexural strength, flexural modulus, and impact strength of the unmodified biocomposite were 53.25 MPa, 3845.82 MPa, and 21.77 kJ/cm^2^. Before modification, the poor interfacial adhesion induced cavities at the interface between PLA and SB. Filled with modified SB, however, the biocomposite possessed obviously increased flexural strength, flexural modulus, and impact strength; for Mg-SB/PLA, these values were 81.82 MPa, 5683.51 MPa, and 34.56 kJ/cm^2^, showing increases of 53.65%, 47.78%, and 58.75% on those of SB-30/PLA, respectively. For Na-SB/PLA, they were increased by 81.03%, 40.79%, and 51.40%, respectively. The reason for this remarkable enhancement was believed to be the compatibilizer role played by the modified fiber. After modification with inorganic salt, a complex compound was produced between the metal cation in the salt and the oxygen atom in the hydroxyl groups in the biomass material; the intermolecular hydrogen bond was destroyed because of the hydroxyl groups’ interaction within the SB fiber. Consequently, the SB surface became rougher, as evidenced in Figure 8, enlarging the interface between the fiber and the resin, and a better bonded interfacial layer formed; the stress could thus be dissipated and conveyed more easily when the biocomposite was loaded, and the interaction between SB and PLA was strengthened, leading to a further improvement in the mechanical properties [18].

**Figure 11:**
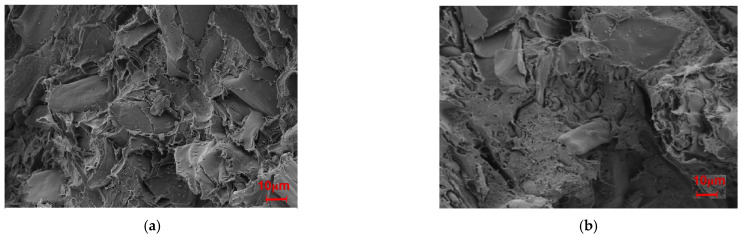
SEM images of the modified biocomposites: (**a**) Mg-SB/PLA and (**b**) Na-SB/PLA.

Figure 11 presents the fracture surfaces of Mg-SB/PLA and Na-SB/PLA. Compared with that of SB-30/PLA (Figure 2c), the fiber was wrapped more tightly by PLA in Figure 11; in addition, the cross-sectional surfaces became much rougher, indicating that the sample was toughened after SB modification, which is consistent with the result shown in Table 3. Further tests indicated that Mg-SB/PLA had a greater impact strength (Table 3) and a much rougher fracture surface (Figure 11) than Na-SB/PLA, meaning that modification with MgSO_4_ helped to improve the flexibility of the biocomposite.

#### 2.2.4. Effects of SB Modification on the Thermal Stability of the Biocomposites

**Figure 12:**
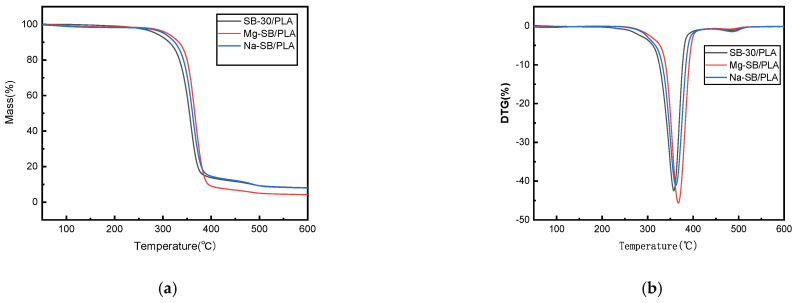
TGA (**a**) and DTG (**b**) curves of the SB-30/PLA, Mg-SB/PLA, and Na-SB/PLA composites.

The thermal stability and degradation properties of Mg-SB/PLA and Na-SB/PLA were measured via a thermogravimetric analysis (TGA). The TGA and DTG curves are presented in Figure 12 alongside the curves of SB-30/PLA as a reference. From the figure, minor weight loss could be observed in the temperature range of 30–150 °C for each biocomposite, potentially due to the evaporation of moisture from the surfaces of the samples. Weight was lost mainly between 200 and 400 °C, and sudden weight loss was seen above 300 °C in all types of biocomposites due to the degradation of cellulose [6]. The corresponding thermal degradation data in this stage and the char residue at 600 °C are summarized in Table 4, showing that the onset temperatures of Mg-SB/PLA and Na-SB/PLA were both higher than that of SB/PLA; furthermore, the peak temperatures also increased, indicating that the modification of SB could improve the thermal stability of the SB/PLA biocomposite, especially for Mg-SB/PLA, with a T_i_ and T_p_ of 6.56 °C and 4.31 °C, correspondingly, the values of which are higher than those of Na-SB/PLA. Therefore, modification with MgSO_4_ was more beneficial for improving the thermal stability of SB/PLA than Na_2_SO_4_ modification.

#### 2.2.5. Effects of SB Modification on the Melt and Crystallization Behavior of the Biocomposites

**Figure 13:**
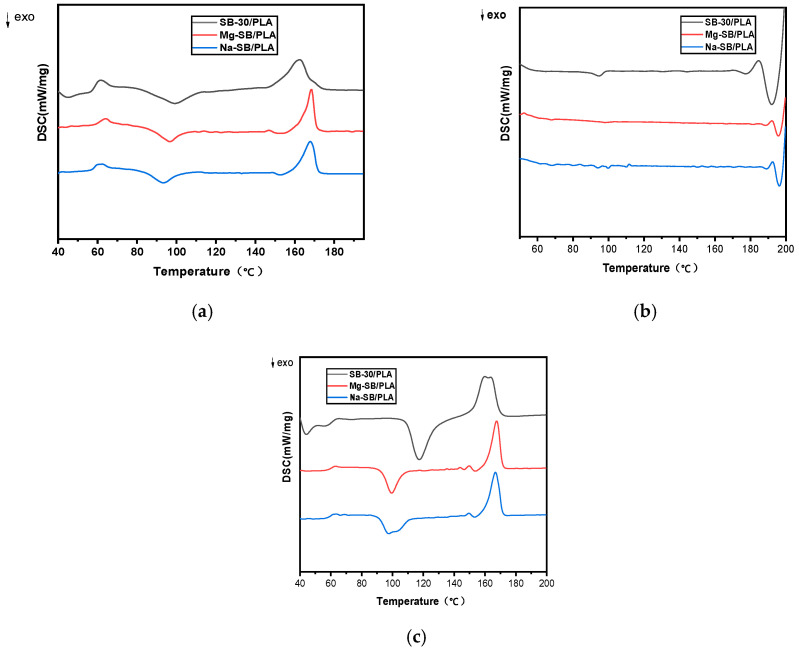
DSC curves of the unmodified and modified biocomposites: (**a**) first heating; (**b**) cooling; (**c**) second heating.

Figure 13 presents the DSC curves of various biocomposites, and the resulting characteristic temperatures are outlined in Table 5.

As shown in Table 5, the effect of SB modification on the glass transition temperature (T_g_) was not very strong, while the enthalpy changes upon the melting of PLA(ΔH_m_) increased obviously, showing that the inherent crystalline region became quantitatively larger [35]; furthermore, the cold crystallization temperature (T_cc_) was found to have decreased after SB modification, suggesting that the inorganic modification affected the crystallization rate of PLA. Both the increase in ΔH_m_ and the decrease in T_cc_ might be due to the improved nucleation effect of SB on the biocomposites after its modification. As discussed before, SB could be distributed much better in the resin after its modification, and more heterogeneous nucleation sites were produced, resulting in increased crystallinity from 4.28% to 5.22% for SB-30/PLA, an increase of 21.96% for Mg-SB/PLA, and an increase to 10.15% (i.e., by 137.15%) for Na-SB/PLA.

#### 2.2.6. Effects of SB Modification on the Hydrophobicity of the Biocomposites

**Figure 14:**
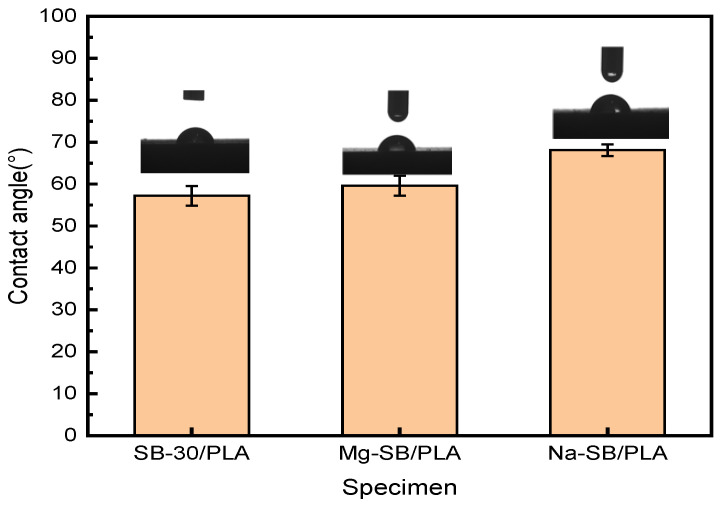
Contact angle (°) of unmodified and modified biocomposites.

The surface wettability of the different biocomposites was examined through their water contact angles, as illustrated in Figure 14.

The water contact angle of SB-30/PLA was found to be approximately 57.2°, confirming its hydrophilic nature. Compared with SB-30/PLA, Mg-SB/PLA and Na-SB/PLA showed increases in their contact angle of 4.20% and 19.05%, respectively. After modification, the amount of -OH groups decreased, as evidenced in the FTIR analysis, which reduced the affinity of the modified SB with water. Furthermore, the modified SB was distributed more uniformly in the PLA; as a result, their interfacial interaction improved, SB was wrapped more tightly by the matrix, and the surface polarity was reduced. The combined effect of this decreased affinity with water and this enhanced interfacial interaction between the fiber and the matrix eventually led to improved hydrophobicity and, thus, increased water contact angles.

#### 2.2.7. Effects of SB Modification on the Water Absorption Behavior of the Biocomposites

**Figure 15:**
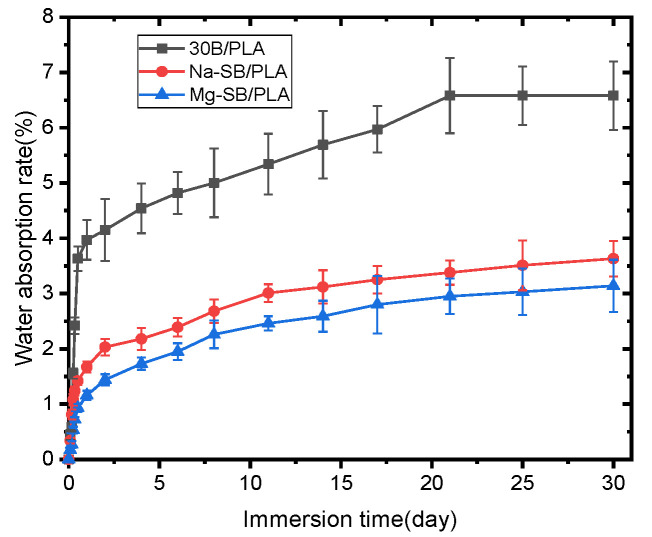
Water absorption of unmodified and modified biocomposites.

The influence of SB modification on the percentages of water absorption over soaking time is illustrated in Figure 15.

**Figure 16:**
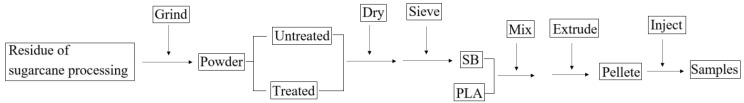
Flowchart for the preparation of biocomposites.

As seen in the figure, all the samples absorbed water quickly and almost linearly with soaking time during the first stage; when extending the soaking time, water absorption increased and then the saturation stage was reached. Similar trends for water absorption have been observed on some other composites, such as sugarcane bagasse nanocellulose/PLA [6] and sugarcane bagasse/epoxy [30] composites. Figure 16 also reveals that the samples became much more water absorption-resistant after SB was modified, with both Mg-SB/PLA and Na-SB/PLA absorbing significantly less water than SB/PLA at any immersion stage; this difference became greater with a longer soaking time. After being soaked in water for 30 days, the water absorption percentages of Mg-SB/PLA and Na-SB/PLA were 3.14% and 3.23%, showing a reduction by 52.28% and 50.91%, respectively, from that of SB-30/PLA, perhaps because the modification of SB with inorganic salts led to a decreased amount of -OH groups in the fibers, as evidenced in the FTIR analysis. The difference in the polarity between SB and PLA was accordingly reduced, and the interfacial compatibility between the reinforcement and the matrix was improved, leading to a compact composite. According to reports from Azka et al. [7], water can be absorbed by biocomposites through various paths, such as entering the available space within the polymer and being absorbed via capillary action along the fiber, whereas these paths are reduced significantly by the compact structure of a biocomposite after modification; consequently, less water will diffuse through the material. The least amount of water absorbed by Mg-SB/PLA may indicate the best interfacial bonding between the filler and the matrix in the biocomposite.

### 2.3. Cost Analysis

As mentioned before, SB-30/PLA had the best overall performance among all the biocomposites. Importantly, SB-30/PLA has a lower material cost than pure PLA.

The current market prices of PLA and SB in China are about USD 480/ton and USD 40/ton, respectively. The material cost of SB-30/PLA would thus be USD 348/ton, representing a reduction of about 27.5% in cost. This reduced material cost may make the biocomposite more competitive on the market.

## 3. Experiments

### 3.1. Materials and Reagents

PLA pellets (3052D, American Nature Works Co., Minnetonka, MN, USA) were purchased from Huizhoushi Tingkai Plastics Co., Ltd. (Huizhou, Guangdong, China); their density was 1.24 g cm^−3^, their glassy transition temperature (T_g_) was between 55 °C and 60 °C, and their melting temperature (T_m_) was between 145 °C and 160 °C. SB was locally collected from Suojincun Market (Nanjing, Jiangsu, China) and then sieved. Sodium sulfate (Na_2_SO_4_, CP) and magnesium sulfate (MgSO_4_, CP) were both purchased from Shanghai Lingfeng Chemicals Reagents Co., Ltd. (Shanghai, China).

### 3.2. SB Modification

The SB was dried at 105 °C for 24 h; a 1 wt % sodium sulfate solution was prepared by mixing sodium sulfate and distilled water; and then, the dried SB (the mass ratio of SB to the sodium sulfate solution was 1:100) was immersed in the solution and stirred homogeneously. After 30 min, the SB was taken out from the solution and washed until the eluent was neutral. After that, the SB was dried at 105 °C until its mass became constant, and sodium sulfate-treated SB was obtained and named Na-SB. The magnesium sulfate-treated SB was obtained similarly and named Mg-SB.

### 3.3. Preparation of SB/PLA Biocomposites

The samples were prepared according to the following flowchart (Figure 16).

The materials were initially prepared in the right quantities and placed in a laboratory oven to dehydrate before being mixed. Separate mixtures were prepared for each biocomposite with different SB contents, as listed in Table 1. The mixtures were supplied individually into a twin-screw extruder (SHJ-25, Nanjing Hongjiayuan Machinery Co., Ltd., Nanjing, China), melted, extruded, and pelletized. The extrusion temperature was set to 170–180 °C, and the rotating speed of the screw was kept at 50 r/min. Then, the pellet was delivered to an injector (CWI-90BV, Shanghai Century-win Machinery Co., Ltd., Shanghai, China) and injected into the samples for tests, the injection temperature was set to 155–165 °C, and the injection pressure was 140 MPa.

Next, each kind of modified SB was used for reinforcement, and their corresponding biocomposites, containing 30 wt % filler, were prepared following the aforementioned diagram of SB/PLA biocomposite preparation. The sample ID and composition of each modified biocomposite are provided in Table 6.

### 3.4. Testing and Characterization

#### 3.4.1. Fourier-Transform Infrared Spectroscopy (FTIR) Analysis

Infrared spectra were acquired using a VERTEX 70 spectrometer (Bruker Optics, Ettlingen, Germany) equipped with a diamond crystal ATR reflection accessory. A spectral analysis was conducted over the wavelength range of 500–4000 cm^−1^ with a resolution of 4 cm^−1^ with 32 scans on the spectrum. Before the measurements were performed, the background spectrum was recorded. The analysis was carried out by pressing the sample against the crystal using a pressure clamp. Before the analysis was performed, the SB or modified SB was mixed with potassium bromide at a mass ratio of 1:100 and then compressed into tablets.

#### 3.4.2. Mechanical Characterization

The quasi-static tensile and flexural characterization was performed using a universal testing machine (E44.304, MTS Industrial Systems (China) Co., Ltd., Shenzhen, China) at room temperature, and the tensile and flexural tests were carried out following the standard test methods ASTM D 638-2010 [41] and ASTM D 790-2010 [42], respectively, with tensile and bending velocities of 50 mm/min and 5 mm/min, in that order.

The Izod impact test with a notch was conducted according to the Chinese National Standard GB/T 1043.1-2008 [43] on an impact testing machine (XJC-25D, Chengde Precision Testing Machine Co., Ltd., Chengde, China).

The values and standard deviations for the tensile, flexural, and impact results were evaluated as the average of five specimens.

#### 3.4.3. Morphology Observation

The morphology of the SB fiber and the fractured surfaces of the biocomposites were obtained on a field-emission scanning electron microscope (SEM) (Merlin Compact, Zeiss Corporation, Oberkochen, Germany) at an accelerating voltage of 3 kV.

#### 3.4.4. Thermal Stability Assessment

Thermal stability was assessed using a thermogravimeter (TG209F1, NETZSCH Gerätebau GmbH, Selb, Germany) under a nitrogen atmosphere. Approximately 5 mg of the crushed material was placed in open alumina crucibles and subjected to pyrolysis from 30 to 600 °C, with a heating rate of 10 K/min. TGA curves and derivatograms (DTGs) were recorded and analyzed using NETZSCH software (TG209F1, NETZSCH-Gerätebau GmbH). The initial degradation temperature (T_i_), which corresponded to the temperature at a 5 wt % mass loss rate on the TGA curve, and the maximum degradation temperature (T_p_), which represented the peak temperature on the DTG curve, were detected to distinguish differences arising from SB content and modification.

#### 3.4.5. Melting and Crystallization Behavior Analysis

The melting and crystallization properties were investigated via differential scanning calorimetry (DSC). The characterization was carried out in a nitrogen atmosphere using a NETZSCH DSC214 (NETZSCH-Gerätebau GmbH, Selb, Germany). Prior to testing, approximately 5 mg of the sample was weighed and placed in an aluminum pan and sealed with an aluminum cover. After stabilization at 20 °C for 5 min, the specimen was heated up to 220 °C at a heating rate of 10 °C/min and kept at a constant temperature of 200 °C for 5 min to delete the thermal history, remove any residual moisture, and fill any voids. Then, the sample was cooled down to room temperature and reheated to 220 °C. The transition temperatures and heat capacities were calculated via NETZSCH analysis software (Proteus70). Equation (1) was used to calculate the crystallinity ($x_{c}$) of PLA [2,15]:

(1) $$x_{c}=\frac{\left|\Delta H_{m}+\Delta H_{cc}\right|}{\omega \Delta H^{\theta }}\times 100\%$$

where $x_{c}$ represents the degree of crystallinity of the sample; $\Delta H_{m}$ and $\Delta H_{cc}$ are the enthalpies of fusion and cold recrystallisation of the specimen, respectively; and $\Delta H^{\theta }$ is the enthalpy of fusion for 100% crystalline PLA, taken from the literature to be 93.6 J/g [44]. ω stands for the PLA mass fraction obtained from the TGA and normalizes the result when considering the percentage of SB particles in the material.

#### 3.4.6. Wettability Testing

The contact angle (θ) of distilled water drops on the surface of each specimen was measured at room temperature using a contact angle instrument (DSA100; KRÜSS GmbH, Borsteler Chaussee, Hamburg, Germany). A 5 µL droplet of distilled water was dropped onto the surface and left for 15 s, and then, the θ values were read at various points. Five specimens were tested for each biocomposite material, and the average contact angles for each sample were recorded.

#### 3.4.7. Water Absorption

The specimens, with dimensions of 130 mm × 12 mm × 6 mm (length × width × depth), were dried to a constant weight and then immersed in distilled water at room temperature. The samples were removed from the distilled water and wiped with tissue paper to remove any leftover surface water. After that, the samples were weighed on a balance to a precision of 1 mg. The weight change for each sample before and after water absorption was recorded. The percentage change at any time (t) as a result of water absorption ($w_{a}$) was determined using Equation (2):

(2) $$w_{a}(\%)=\frac{m_{t}-m_{0}}{m_{0}}\times 100$$

where $w_{a}$ stands for water absorption (%), and $m_{t}$ and $m_{0}$ denote the weights of the samples (g) before and after exposure to water absorption, respectively. The average value from at least five measurements was calculated to obtain the reported results.

## 4. Conclusions

In this study, SB and PLA were blended using an extrusion process and then pelletized, followed by an injection molding process to prepare the SB/PLA biocomposite samples. The effect of the dosage of SB and its modification with inorganic salt on the properties of the biocomposites was investigated. The following are the main conclusions drawn from this research.

The addition of SB had a negative effect on the mechanical properties and thermal stability of PLA. Nonetheless, the samples containing 30 wt % SB had the greatest tensile strength, tensile modulus, flexural strength, and crystallinity among all the SB/PLA biocomposites. Though an increased dosage of SB in the biocomposite would lead to a gradual increase in water absorption and weakness in thermal stability, SB-30/PLA had adequate water uptake and thermal stability when compared with the other samples. Generally, SB-30/PLA had the best comprehensive performances among all the SB/PLA biocomposites. Additionally, 30 wt % PLA was replaced with SB in SB-30/PLA, leading to a significant reduction in cost of about 27.5%; this consequently enhanced the market competitiveness of the sample.

The inorganic salt modification of SB with Na_2_SO_4_ or MgSO_4_, provided for the first time in this study, not only improved the mechanical properties and thermal stability of the SB/PLA biocomposite significantly but also increased its crystallinity and hydrophobicity. In addition, water absorption was greatly reduced. All these factors demonstrated the significant effect of SB modification, especially with MgSO_4_, on the physico-mechanical properties of SB/PLA biocomposites, which could broaden the applicability of PLA-based biocomposites and yield a material with a reasonable combination of properties for applications in packaging, decoration, and other areas.

In the future, properties might be improved even further with the optimization of the dimensions of the bagasse fibers.

## Figures and Tables

**Table 1:** Main thermal degradation parameters of PLA and SB/PLA.

Sample ID	T_i_/°C	T_p_/°C	Char Residue/wt%
PLA	347.84	382.53	0.99
SB-10/PLA	312.23	369.32	3.64
SB-20/PLA	285.61	359.44	2.95
SB-30/PLA	284.33	358.56	8.37
SB-40/PLA	269.78	358.22	12.32
SB-50/PLA	260.79	358.03	12.91

**Table 2:** The DSC testing outcomes of PLA and SB/PLA biocomposites.

Sample ID	T_g_/°C	T_cc_/°C	ΔH_c_ (J/g)	T_m_/°C	ΔH_m_ (J/g)	X_c_/%
PLA	58.9	130.63	−38.22	159.12	39.53	1.39
SB-10/PLA	58.5	123.60	−35.35	155.86	42.21	8.14
SB-20/PLA	58.4	127.21	−26.41	157.93	35.58	12.25
SB-30/PLA	58.6	128.20	−18.51	159.01	29.52	16.8
SB-40/PLA	58.9	128.02	−16.37	158.02	22.79	11.4
SB-50/PLA	59.6	125.41	−17.22	156.39	20.96	7.99

**Table 3:** Mechanical properties of the biocomposites before and after SB modification.

Sample ID	Flexural Strength (MPa)	Flexural Modulus (MPa)	Impact Strength (kJ/cm^2^)
Strength	42.04 ± 4.87	3845.82 ± 269.52	21.77 ± 3.49
Mg-SB/PLA	81.82 ± 5.71	5683.51 ± 376.16	34.56 ± 4.86
Na-SB/PLA	96.4 ± 3.46	5414.59 ± 450.32	32.96 ± 4.02

**Table 4:** Main thermal degradation parameters of the SB-30/PLA, Mg-SB/PLA, and Na-SB/PLA biocomposites.

Sample ID	T_i_/°C	T_p_/°C	Char Residue/wt%
SB-30/PLA	284.33	358.56	8.37
Mg-SB/PLA	319.05	365.88	2.89
Na-SB/PLA	312.49	361.57	7.55

**Table 5:** DSC analysis parameters of the SB-30/PLA, Mg-SB/PLA, and Na-SB/PLA biocomposites.

Sample	T_g_/°C	T_cc_/°C	ΔH_c_ (J/g)	T_m_/°C	ΔH_m_ (J/g)	X_c_/%
SB-30/PLA	58.6	116.9	−18.51	156.7	22.52	4.28
Mg-SB/PLA	60.3	99.5	−26.81	167.8	30.23	5.22
Na-SB/PLA	59.0	97.7	−28.13	167.0	34.78	10.15

**Table 6:** SB/PLA biocomposite formulation and rates.

Sample Codes	PLA	SB-10/PLA	SB-20/PLA	SB-30/PLA	SB-40/PLA	SB-50/PLA	Na-SB/PLA	Mg-SB/PLA
PLA/wt %	100	90	80	70	60	50	70	70
SB/wt %		10	20	30	40	50		
Na-SB/wt %							30	
Mg-SB/wt %								30

## Data Availability

The original contributions presented in this study are included in the article. Further inquiries can be directed to the corresponding author.
