# Correction: Peng et al. Effect of Sugarcane Bagasse Content and Modification on the Properties of Sugarcane Bagasse/Poly(lactic Acid) Biocomposites. *Molecules* 2025, *30*, 1583

**DOI:** 10.3390/molecules30244720

**Published:** 2025-12-10

**Authors:** Youxue Peng, Wen Lei, Wangwang Yu, Yong Chen

**Affiliations:** 1 College of Science, Nanjing Forestry University, Nanjing 210037, China; 18801581152@163.com (Y.P.); chenyongpoly@163.com (Y.C.); 2 School of Mechanical Engineering, Nanjing Vocational University of Industry Technology, Nanjing 210023, China; yuww@niit.edu.cn

## Error in Figure/Table

In the original publication [1], there was a mistake in “Table 2. The DSC testing outcomes of PLA and SB/PLA biocomposites”, “Table 4. Main thermal degradation parameters of the SB-30/PLA, Mg-SB/PLA, and Na-SB/PLA biocomposites”, and “Table 5. DSC analysis parameters of the SB-30/PLA, Mg-SB/PLA, and Na-SB/PLA biocomposites” as published.

The T_cc_ values shown in Table 2 were misread from the DSC curves (Figure 4); the correct values should be 119.9 °C for PLA, 113.3 °C for SB-10/PLA, 115.3 °C for SB-20/PLA, 116.9 °C for SB-30/PLA, 116.8 °C for SB-40/PLA, and 114.9 °C for SB-50/PLA.

Owing to the correction of the T_cc_ values in Table 2, some explanation in the Section “2.1.3. Effects of SB Content on the Melt and Crystallization Behavior of the Biocomposites” has been modified accordingly; i.e., the description of “…, and SB-30/PLA had the greatest T_cc_ value of 128.20 °C and a T_m_ value of 159.01 °C.” was corrected to “…, and SB-30/PLA had the greatest T_cc_ value of 116.9 °C and a T_m_ value of 159.01 °C.”

The X_c_ value is calculated by Equation (1); the X_c_ value of SB-30/PLA in Table 5 was recorded as 4.28% due to miscalculation, and the result has been corrected to 16.8% after a correct calculation. Meanwhile, the T_m_ and ΔH_m_ values of SB-30/PLA in Table 5 were recorded as 156.7 °C and 22.52 J/g because of the clerical error; they have been corrected to 159.0 °C and 29.52 J/g.

Owing to the correction of the X_c_ value in Table 5, some explanation in the Section “2.2.3. Effects of SB Modification on the Melt and Crystallization Behavior of the Biocomposites” was modified accordingly; i.e., the description of “As discussed before, SB could be distributed much better in the resin after its modification, and more heterogeneous nucleation sites were produced, resulting in increased crystallinity from 4.28% to 5.22% for SB-30/PLA, an increase of 21.96% for Mg-SB/PLA, and an increase to 10.15% (i.e., by 137.15%) for Na-SB/PLA.” has been deleted, and, accordingly, the description of “…increases in the flexural strength, flexural modulus, impact strength, and crystallinity of SB-30/PLA” in the abstract has been corrected to “…increases in the flexural strength, flexural modulus, and impact strength of SB-30/PLA”.

The T_i_ values of Mg-SB/PLA and Na-SB/PLA, as well as the char residue of Mg-SB/PLA in Table 4, were misread from Figure 12; they have now been corrected to be 309.05 °C, 302.07 °C, and 4.18%, respectively. Accordingly, the description of “with an increased T_i_ and T_p_ of 6.56 °C and 4.31 °C, correspondingly” in Section “2.2.4. Effects of SB Modification on the Thermal Stability of the Biocomposites” has been corrected as “with an increased T_i_ and T_p_ of 24.72 °C and 7.32 °C, correspondingly”.

Figure 16 illustrates the flowchart for the preparation of biocomposites. It was moved from Section “2 Results and discussion” to Section “3 Experimentals”.

The authors state that the scientific conclusions are unaffected. This correction was approved by the Academic Editor. The original publication has also been updated.
